# LGBTQ+ Youth Health: An Unmet Need in Pediatrics

**DOI:** 10.3390/children9071027

**Published:** 2022-07-11

**Authors:** Pierluigi Diana, Susanna Esposito

**Affiliations:** Pediatric Clinic, Department of Medicine and Surgery, University of Parma, 43126 Parma, Italy; pierluigidiana@yahoo.it

**Keywords:** adolescents, lesbian, gay, LGBTQ+, pediatric population, sexual and gender minorities, transgender

## Abstract

At present, lesbian, gay, bisexual, transgender, queer and intersex people (LGBTQ+) are increasingly being empowered to freely express themselves, particularly young people and rising generations. Although data underline the trend of more open expression of different sexual orientations and gender identities, LGBTQ+ adolescents still suffer discrimination in the health care framework. Inclusive care by providers to look after the health of LGBTQ+ indiviuals is needed. Pediatricians are often the first health care providers for LGBTQ+ youth facing their sexual and gender identities. Unfortunately, pediatricians have limited knowledge about LGBTQ+ issues, which keeps them from fulfilling the specific needs of LGBTQ+ youth. The purpose of this review is to frame the most important aspects of LGBTQ+ youths’ lives, including risks, difficulties and needs, that pediatricians should investigate and meet to provide these youth with better and more individualizedassistance regarding their health. A literature analysis showed that pediatricians have insufficient knowledge of and comfort with several items regarding the management of LGBTQ+ youths. Increased awareness and knowledge of the specific and exclusive needs of LGBTQ+ adolescents are mandatory, including dedicated pediatric LGBTQ+ health care training. This would give them the opportunity to forward an inclusive health care system, thus reducing the risks related to stigma, bullying and family rejection and promoting sex education. Further studies are needed to better evaluate the prevalence of LGBTQ+ youths, gender-based medicine in pediatrics and the effects of COVID-19 on the LGBTQ+ younth population due to increased risks of psychosocial suffering, isolation and mental diseases.

## 1. Introduction

At present, lesbian, gay, bisexual, transgender, queer and intersex people (LGBTQ+) are increasingly being empowered to freely express themselves, particularly young people and rising generations [1]. It is evaluated that the proportion of sexual and gender minorities (SGM) in the entire population stands at around 3.8% [2]. National surveys conducted in the United States (US) in 2020 estimated that almost 9% of adolescents between 13 and 17 years of age recognize themselves to be lesbian, gay or bisexual, and 0.73% identify as transgender, although a higher prevalence is possible [3]. Regarding the population between ages 9 and 12 years, it has been shown that approximately 1%–3% identify as gay or lesbian, 3%–5% identify as bisexual, and 1%–5% are unsure about their sexual orientation [4]. Italy still lacks data on sexual identity and orientation among adolescents. The latest ISTAT census in 2011 showed that 2.4% of the population self-identify as SGM, especially among young people, but no mention was made of the population under 18 years of age [5].

Although data underline the trend of more open expression of different sexual orientations and gender identities, LGBTQ+ adolescents still suffer discrimination in the health care framework. Inclusive care by providers to look after their health is needed. [6,7,8,9,10].

Pediatricians are often the first physicians in the health care system to care for LGBTQ+ adolescents facing their sexual and gender identities. Unfortunately, as shown by several authors, pediatricians have insufficient knowledge of LGBTQ+ issues [11,12,13,14], which keeps them from fulfilling the specific needs of their patients. The purpose of this review is to frame LGBTQ+ youths’ lives, including risks, difficulties and needs, that pediatricians should investigate and meet to provide these youth with better and more individualized assistance regarding their health. This is a narrative review of the literature on LGBTQ+ youths’ care in pediatrics, carried out by the Pediatric Clinic at the University of Parma, Parma, Italy. Systematic searches were performed in PubMed up until March 2022. The language was restricted to English. Search terms included “LGBTQ”, “LGBTQ+”, “Transgender”, “Gender Non-conforming”, “Non-Binary” and “Health” in combination with the words adolescents, children and/or youth. Original research studies, review articles, letters to the editor and cohort studies published between 2010 and 2022 were included. Data from earlier studies were taken into account if relevant to the scope of this review. All relevant articles were then evaluated, and pertinent articles were included in this review. Since LGBTQ-related glossary is fluid and may be subject to change from time to time, Table 1 provides an overview of terms to serve as a guide for this review. This represents a starting point for discussion because terms and definitions are evolving and changing and can have different meanings to different people within the LGBTQ+ community.

## 2. Gender Identity

Gender identity represents a personal inner concept of self as male, female, both or neither. It is the perception of themselves and what/how they define themselves [15,16,17,18]. It usually develops in early childhood, through a dynamic process from childhood to adolescence and adulthood, so the gender identity of a pre-pubertal child may also change later in life [15]. Conversely, the gender identity developed during puberty likely anticipates the adult gender identity [16]. Gender identity and related expressions differentiate from sexual orientation [17], which is the inherent sentimental or sexual attraction to other individuals, independent of one’s gender identity [18].

Transgender youth, in some but not all cases, may pursue gender-affirming therapies such as hormonal therapy and surgery. The risks and benefits of these treatments have been extensively studied in adults, whereas studies in adolescents are scarce [19,20]. For a long time, children and adolescents suffering from gender dysphoria were offered psychotherapeutic treatments with the aim of making them identify with their sex assigned at birth. These therapeutic efforts have been demonstrated not to work [21]. The use of hormonal therapies in transgender minors commenced in the 1980s in the Netherlands. Currently, gender-affirming interventions in transgender adolescents are conceptualized in three steps: puberty suppression, gender-affirming hormones and gender-affirming surgery. Several guidelines are now available and help clinicians in the decision-making process with a focus on the age at which these young individuals are likely to properly understand and consent to these treatments [22,23,24]. According to the Endocrine Society, before starting hormonal therapy, a qualified mental health practitioner must confirm the presence of persistent gender dysphoria and that the adolescent has a stable social and medical environment [22]. Moreover, a pediatric endocrinologist must exclude medical contraindications.

With regards to gender-affirming surgery, it is usually performed when the individual is 18 years old or older. It may consist of bilateral mastectomy and phalloplasty in transgender men whereas transgender women may undergo facial feminization procedures and vaginoplasty. It is important to highlight that gender-affirming therapies in transgender youth are associated with mental health benefits and a better quality of life [25].

Gender identity development in children born with differences in sex development (DSD) is another major issue. There is no consensus among the scientific community about the correct timing for sex-assignment surgery in individuals with DSD since they could develop a different gender identity after adolescence [26,27,28]. The assumption that sex-assignment surgery will guide a child’s future gender identity has been demonstrated to be wrong [29,30]. Therefore, a more prudent approach will be to let the eventual stable gender identity in a child or adolescent with DSD guide and determine the sex assignment.

## 3. Home and Family

LGBTQ+ adolescents may experience conflict within the context of home, parenting and family. Parental awareness of their child’s gender identification and an evaluation of their support should be one of the first steps of a pediatrician when caring for an adolescent [15]. In their cross-sectional survey conducted among 733 LGBTQ+ youth aged 13–21 years, Hoffman et al. highlighted how responders cite family issues as critical worries to discuss with physicians [31]. When families accept LGBTQ+ members, their life satisfaction improves and depressive symptoms decrease [32,33]. Their sexual practices become safer too; Wilson EC et al. showed a positive association between condom use and parental support among transgender female adolescents [34].

The family rejection that LGBTQ+ individuals may experience could lead them to seek approval, sharing and love from chosen families. A chosen family is a group of people who are emotionally close to one another and consider each other family without any biological or legal bonds [35]. There is still a lack of data about this topic as it relates to pediatrics, but in a recent study conducted on an adult population, Levin et al. reported the experience of Tish, a nonbinary person who identifies as pansexual who met a chosen family for the first time when as a teenager and no longer keeps a relationship with the biological family [36].

Zelin et al. underlined how 45% of pediatric residents may still lack knowledge about sexuality and gender identity among the younger population and 6% would still be afraid of hurting their parents with conversations focused on sexuality and gender identity [37]. In addition, Kitts et al. found that only 29% of pediatric providers routinely approach topics about sexual orientation and only 8.5% approach topics about gender identity with sexually active adolescents [12].

As already emphasized, it is crucial for medical providers to share a message of safety and inclusion with patients and their families [38]. A pediatrician should allow all the family members to freely express their feelings and their thoughts, finding time and space to engage in a conversation with the patient and the family one at a time. Gender-affirming terminology and interventions should be explained to families, including helping them to connect with supportive organizations. It can be normal for family members to be anxious about their children, particularly because of societal discrimination and stigma, given their different way of expressing or feeling about themselves [38]. Some families may feel insecure about their child’s future, others may not be inclusive due to personal values, and others may experience loneliness and sadness [39].

Medical providers should stay calm and non-judgmental, and promote private discussions about fears and feelings to facilitate communication between all family actors. Misinformation obtained from the internet, social media, confidants or others should be investigated, and the lack of information should be overcome by providing education through research articles and websites, LGBTQ+ organizations, and discussions on gender-affirming medical and non-medical interventions for youth that experience gender dysphoria [39].

## 4. School and Bullying

School is a critical social environment for youth. LGBTQ+ students have increased risk for bullying at school in comparison with their non-LGBTQ+ peers (34.2% vs. 18.8%, respectively) [4,40]. In a 2018 regression analysis, Baams showed that lesbian, gay, bisexual and questioning adolescents presented profiles characterized by polyvictimization and psychological and/or physical abuse with a higher frequency than profiles without adversity [41]. Youth who are bullied over and over again by peers experience several problems: they may internalize verbal and physical abuse by blaming themselves; they may develop anxiety and depression; or they may suffer from stomachaches and headaches, or other psychosomatic conditions [42,43]. Bullied gender non-conforming adolescents are also at a higher risk of drug abuse [40]; moreover, LGBTQ+ bullying effects last into adulthood [44]. It is well suggested in recent research that bullying based on stigma is more closely tied to health problems than bullying in general [45]. Additionally, a 2019 American study published in the Journal of Adolescent Health shows that LGBTQ-inclusive sex education correlates with a lower rate of school-based victimization and adverse mental health, demonstrating that inclusive sex education may enhance school climate and psychophysical health for youth [46]. In fact, when antibullying policies, including sexual orientation policies, are provided in schools, LGBTQ+ youths have a lower risk of suicide attempts [47].

## 5. Sexual Health and Education

Asking questions about and counseling on items related to sexual and reproductive health, including sexual orientation/behavior and gender identity, are essential. Some studies have shown that LGBTQ+ young individuals face dangerous early sexual experiences to a greater extent than their heterosexual peers (65.5% vs. 35.8%), including a higher risk of having sex with unknown partners, having sex while abusing drugs, having unprotected sex during their last intercourse, and not undergoing tests for sexually transmitted infections (STIs) [4,48,49,50]. This also seems to be related to poor inclusive sexual education. Renold et al. highlighted that, in the United Kingdom, less than 20% of LGBTQ+ adolescents received sex education related to LGBTQ+ issues [51]. In a survey conducted in 2017, transgender students declared having sexual intercourse before turning 13 years old, having four or more sexual partners, using drugs or alcohol before sex, and using fewer condoms (than cisgender students) [52]. Ristori et al. recruited 50 Italian transgender adolescents aged 11–18 years in a cross-sectional study to explore the experiences of these Italian TGN youth in relation to sexual matters. Their research showed a direct correlation between having sexual intercourse before the age of 14 and smoke/drug consumption, thus underling how a negative sexual (and health) outcome could be prevented by improving gender-inclusive sexual education [53]. In Italy, both parents and school providers still experience difficulty in addressing sex education in adolescents [54]; therefore, the Italian National Institute of Health has recently affirmed that a National Plan for sexual health promotion and STI prevention is needed [55]. In view of this, pediatricians should screen for STIs among LGBTQ+ youths in order to reduce patient morbidity and to prevent HIV and/or secondary STI transmission [56]. It has been shown that adolescent men who have sex with men (MSM) present increased rates of gonorrhea, *C. trachomatis* and syphilis compared to their heterosexual peers [57]. Similarly, the HIV infection rate is higher; in 2010, in the US, youth ages 13–29 represented 26% of all new infections, particularly among young MSM [56,58]. Adolescents symptomatic with fever from unknown origins or from a mononucleosis-like syndrome, or with previous high-risk exposure to an STI should be investigated for acute HIV infection (AHI) as 50%–89% of patients with AHI generally manifest symptoms such as fever, sore throat, rash, lymphadenopathy, mucocutaneous ulcers or muscular pain [59]. In 2018, as a response to the expanding urgency to prevent HIV transmission among youth, a fixed-dose combination of tenofovir disoproxil fumarate with emtricitabine was approved in the US as an oral pre-exposure prophylaxis (PrEP) for HIV in adolescents; in 2019, tenofovir alafenamide with emtricitabine was also approved [60,61]; however, data show that only 1.5% of the adolescents at risk for HIV have been prescribed PrEP versus 10% of adults [62,63].

Recent US data showed that, in 2018, the national *Chlamydia* and gonorrhea infection rates for ages 15–19 were 2.11% and 0.4%, respectively [64]. Data related to sexual partners were not reported, but previous studies showed that *C. trachomatis* infection was highly prevalent also among screened adolescent MSM, with rectal infection from *C. trachomatis* reported as 3–10.5% and pharyngeal infection reported as 0.5–2.3% [65,66].

Human papilloma virus (HPV) is also common among LGBTQ+ youths, therefore causing anogenital warts, and cervical and anal dysplasia [56]. It has been evaluated that serotypes 6 and 11 are responsible for 90% of non malignant lesions [67], whereas serotypes 16–18 are mainly responsible for cervical and anal dysplasia. The risk of the progression of cervical and anal dysplasia in adolescents increases when associated with HIV, multiple sexual partners and the presence of external warts [68]. For immunization, a nine-valent HPV vaccine is available and recommended for all young individuals (for girls ages 11–26 and for boys age 11–21) [69].

As Wood et al. underlined, it is also relevant to consider that an increased number of sexual partners/sex is not necessarily the main reason for high STI frequency in the infection rate of LGBTQ+ youths. For MSM, the anatomy and immunology of the rectal mucosa led to a greater susceptibility to STIs and HIV [56]. When collecting a sexual anamnesis, pediatric providers should avoid every type of judgment and opt for using inclusive language, choosing gender-neutral communication and considering that sexual practices do not necessarily depend on sexual orientation ( e.g., both homosexuals and heterosexuals can perform anal sex) [70]. Before performing a physical examination, especially of the genitals, it is important to assess the comfort level of the patient with respect to sensitive phases of the examination. TGNs may feel great discomfort if they still have their natal sexual anatomy; therefore, always discuss every phase of the examination with the patient [56]. Table 2 summarizes the main sexually transmitted infections in LGBTQ+ youths.

## 6. COVID-19 and LGBTQ+ Youths

In the last two years, the Severe Acute Respiratory Syndrome Coronavirus 2 (Sars-Cov-2) pandemichas impacted social and health systems worldwide. It has clearly been shown how deeply COVID-19 negatively impacted youth’s psychological well-being globally [71], but a lack of information about LGBTQ+ youths has been collected. In a recent paper, Ormiston et al. highlighted the expected deeply negative effects of the pandemic on LGBTQ+ youths’ health, reporting that health policies such as lockdowns and school closures with remote learning are likely to worsen the dangers of depression, suicide, drugs abuse and anxiety [72]. It has been reported that more than 50% of SGM young people in the US have experienced increased anxiety or depressive symptoms since the start of the COVID-19 crisis due to isolation from support systems, lack of family endorsement and limited access to health care services [73,74,75,76]. In their study, Fish et al. evaluated 812 COVID-19-related content pages of chat-based support groups for LGBTQ+ youths (ages 13–19) during the pandemic. They report how LGBTQ+ youths appeared to be worried about “being stuck at home with unsupportive parents”; locked up with parents described as super religious and homophobic; and unable to freely express themselves or to access friends, gender and sexuality alliances and supportive staff [77]. Data collected from a cohort of 1031 young people living in the US between November 2020 and December 2020 showed that for 71.5% of SGM youth, the pandemic affected their mental wellness “a lot”, compared to 42.2% of their heterosexual peers [78].

It has also been evaluated that LGBTQ+ people present a greater risk of developing severe COVID-19 due to poorer health care services related to stigma and discrimination [79]. Interestingly, data collected on the adult population showed a higher COVID-19 vaccination rate among LGBTQ+ individuals than heterosexual individuals (85.4% vs. 76.3%) [80].

## 7. The Role of the Pediatrician

Since 1973, homosexuality has been removed as a psychiatric disorder in the DSM. Despite this, misperception and faults are still widespread among health providers, including pediatricians. As Lena et al. observed, pediatricians often show a scarcity of ability to approach LGBTQ+ youth-related topics due to lack of knowledge (47%) and fear of being offensive towards parents or children (32%); 5% believe that “it is inappropriate at this age” [81]. In a later study, 45% of pediatric residents declared having a lack of knowledge; 25% declared not knowing how to ask about sexual orientation; 15% and 6% declared being offensive to patients and parents, respectively; and 10% believed that ”discussion is inappropriate given the age” [37]. Pediatricians take charge of their patients’ health from birth to adolescence, which means they face a crucial time when patients self-identify from a sexual and gender point of view. A recent American Academy of Pediatrics policy statement on transgender youth care highlighted that children who later identified as trans and non-binary declared having felt their gender was ‘different’ at age 8.5 years of age, on average, despite the fact that most did not reveal these feelings until adulthood [46]. A pediatrician should talk with adolescents about their sexual behavior, if possible, not in the presence of the parents [82,83,84]. Meckler et al. showed that 33% of adolescents did not reveal their sexual orientation just because no corresponding question was asked by their doctor [85]. It has been observed that LGBTQ+ youths would rather benefit from spaces and support especially designed for LGBTQ+ individuals [86]. A first imperative measure would be creating an environment that promote LGBTQ+ youths to feel confident with revealing their sexuality and gender identity. This would allow pediatricians to provide precise, potentially needed lifesaving supports, including gender-affirming treatments for TGN youth, mental health care and family involvement [87]. Pediatricians may build a more inclusive interaction with adolescents by escaping questions that presuppose the gender of the patient’s partners and investigating the favored gender pronoun to be used (i.e., he/him, she/her or they/them) [8]. Conversations about sexual orientation and gender identity should always be safeguarded, considering that some adolescents may not have talked about the topic with parents or other people. For bullying concerns, pediatricians can instruct parents and children about how to recognize youth experiencing LGBTQ+ bullying by investigating signs of depression, anxiety, shame and isolation [8]. Inclusive sex education may be provided by pediatric care workers, which can prevent premature sex and sexual risk-taking, and increase condom use [88,89].

Pediatricians should definitively play a leading role in advocating and caring for LGBTQ+ youths and families. Table 3 summarizes the most recent resources and recommendations available for supporting the LGBTQ+ community.

## 8. Conclusions

Pediatricians have limited knowledge about and comfort with many items regarding the care of LGBTQ+ youths. The present review supports the necessity for greater consciousness and knowledge of the specific and exclusive needs of LGBTQ+ adolescents, including dedicated pediatric LGBTQ+ health training. This would provide the opportunity to forward an inclusive health care system, thus reducing risks related to stigma, bullying and family rejection and promoting sex education. Physicians can set the tone of comfort by framing three basic principles of care when approaching a patient: non-judgmental contact, respect and honesty [90]. Our findings suggest the urgency of specific training for pediatric residents and pediatricians in terms of LGBTQ+ care, including how to talk about sexual orientation, sexual attraction, and gender identity while taking a sexual anamnesis from a sexually active adolescent; how to identify specific risks such as depression, suicidal thoughts, transmitted sexual infections; and how to support LGBTQ+ parents and families.

This review highlights that there are extremely limited data regarding the epidemiology of the LGBTQ+ population under 18 years of age. More studies should be performed in order to better estimate the prevalence of LGBTQ+ youths in Italy and in Europe. We also still lack information about gender-based medicine in pediatrics. Further efforts should be directed toward deeply investigating these items in childhood, even focusing on how to care for transgender and non-binary youths when treating birth-assigned sex-related diseases. More attention should also be directed to the effects of COVID-19 on the population of LGBTQ+ youths due to the increased risks of psychosocial suffering, isolation and mental diseases.

## Figures and Tables

**Table 1:** LGBTQ+-related terminology.

Term	Definition
Gender identity	A personal inner perception of self as male/female/both/neither
Transgender	People who cross delineate categories of gender based on their assigned sex at birth
Gender dysphoria	The discontent that comes with the inconsistency between one’s perceived gender and one’s birth sex
Sex assigned at birth	The label assigned by a physician based on external/internal anatomy, chromosomes, etc.
Gender non-conforming people	People who diverge from the expected behavior of their gender in a given society or historical era
Genderqueer	Gender non-conforming people who do not behave in or conform to the binary concept of male or female
Sexual orientation	An inherent sentimental or sexual attraction to other individuals
Cisgender	People who identify themselves with a gender that is consistent with their sex assigned at birth
Gay	A person who is emotionally or sexually attracted to members of the same sex
Lesbian	A woman who is emotionally or sexually attracted to other women
Bisexual	A person who is emotionally or sexually attracted to more than one sex, gender or gender identity
Queer	An umbrella term that refers to a spectrum of gender identities and sexual orientations that do not belong to the mainstream
Intersex	People who are born with a variety of differences in their reproductive anatomy.

**Table 2:** Main sexually transmitted infections in LGBTQ+ youths. PID: pelvic inflammatory disease.

Pathogen	Most Common Symptoms	Most Common Symptoms
*Chlamydia trachomatis*	Watery, scant urethral discharge, dysuria. If epididymitis: unilateral testicular pain, swelling, tenderness. If proctitis: diarrhea, rectal pain/bleeding, tenesmus	Cervicitis, urethritis If PID: abdominal pain, fever, back pain, dyspareunia
*Neisseria gonorrhea*	Urethra discharge, purulent urethritisIf epidimytis or proctitis: same of *Chlamydia trachomatis*	Cervicitis, urethritis If PID: abdominal pain, fever, back pain, dyspareunia
Bacterial vaginosis		Vaginal irritation with copious and malodorous discharge
Syphilis	Primary: solitary painless ulcer (chancre) Secondary: maculopapular rash, cutaneous lesions, lymphadenopathy Tertiary: cardiological involvement, gummatous syphilis, involvement of neurological system	
HIV	Unknown origin fever or mononucleosis-like syndrome Fever, lymphadenopathy, pharyngitis, rash, muscular pain, mucocutaneous ulcers	
HPV	Anogenital warts, cervical dysplasia, anal dysplasia	

**Table 3:** Main resources for the care of LGBTQ+ youths. CHEC: Culturally Effective Pediatric Care.

Topic	Year	Resource
LGBTQ+ youth care	2013	Office-Based Care for Lesbian, Gay, Bisexual, Transgender, and Questioning Youth [91]
TGN youth care	2018	Ensuring Comprehensive Care and Support for Transgender and Gender-Diverse Children and Adolescents [92]
CHEC in pediatrics	2013	Enhancing Pediatric Workforce Diversity and Providing Culturally Effective Pediatric Care: Implications for Practice, Education, and Policy Making [93]
Nondiscrimination in pediatrics	2007	Nondiscrimination in Pediatric Health Care [94]

## Data Availability

Not applicable.
